# Discordance in ER, PR, HER2, and Ki-67 Expression Between Primary and Recurrent/Metastatic Lesions in Patients with Primary Early Stage Breast Cancer and the Clinical Significance: Retrospective Analysis of 75 Cases

**DOI:** 10.3389/pore.2021.599894

**Published:** 2021-04-09

**Authors:** Li Peng, Zhen Zhang, Dachun Zhao, Jialin Zhao, Feng Mao, Qiang Sun

**Affiliations:** ^1^ Department of Breast Surgery, Peking Union Medical College Hospital, Beijing, China; ^2^ Department of Pathology, Peking Union Medical College Hospital, Beijing, China

**Keywords:** biomolecular status, discordance, recurrence, metastatic disease, PFS, breast cancer, surrogate subtypes

## Abstract

**Background:** The objective was to explore the discordance in the expression of the estrogen receptor (ER), progesterone receptor (PR), human epidermal growth factor receptor 2 (HER2), and Ki-67 between primary and recurrent/metastatic lesions in patients with early stage breast cancer as well as the prognostic impact.

**Method:** Patients with early-stage primary breast cancer and confirmed recurrence/metastasis at Peking Union Medical College Hospital between January 2005 and August 2018 were screened. The details of discordance in each parameter between primary and recurrent/metastatic lesions and progression were recorded. Regression and survival analysis were applied to determine the association and clinical impact of the discordance.

**Results:** We evaluated 75 patients. The discordance rate of ER, PR, HER2, and Ki-67 expression was 9.3, 14.7, 14.7, and 21.5%, respectively. Additionally, 66.7, 11.8, 14.3, and 0% of patients with Luminal A, Luminal B, HER2, and triple-negative primary tumors presented with a different subtype for the recurrent/metastatic tumors, respectively. No statistical difference in progression-free survival was observed according to the subtype of the recurrent or metastatic breast cancer (*p* > 0.05). Among 69 patients for whom treatment was adjusted after recurrence or metastasis, 66 patients remained recurrence-free during the follow-up period.

**Conclusion:** For patients with early-stage breast cancer, the ER, PR, HER2, and Ki-67 expression profile for recurrent/metastatic tumors does not always match that of the primary tumor. After adjusting treatment according to the receptor expression in recurrent/metastatic lesions, most patients remained progression-free during the follow-up period.

## Introduction

Although the prognosis of breast cancer is relatively better than that for most malignancies, it remains the second most common cause of death for female cancer patients worldwide [1–3]. However, the exact mechanism by which breast cancer progresses is still unknown [4, 5]. Early detection of breast cancer is universally acknowledged as a critical factor in improving the outcome and survival of patients [6]. One of the prognostic dilemmas is that some patients with early-stage breast cancer who undergo systemic therapy may experience recurrence or metastasis [7].

Researchers have hypothesized that the diversity in prognosis may be due to differences in the biological characteristics of the primary tumor and the recurrent or metastatic tumors [8, 9]. The expression levels of the estrogen receptor (ER), progesterone receptor (PR), human epidermal growth factor receptor 2 (HER-2), and Ki-67 biomarker have been proven to be associated with the prognoses of breast cancer [10, 11]. Additionally, differences in ER, PR, HER2, and Ki-67 expression between the primary tumor and recurrent or metastatic tumors as well as the clinical significance have been reported in several studies [12–16]. However, there is still a lack of corresponding evidence for patients with early-stage breast cancer. As personalized treatment and follow-up care are highly recommended for patients with early-stage breast cancer, the exploration of potential alterations in their biological characteristics is essential [17, 18].

The objective of the present study was to explore the discordance and prognostic impact of ER, PR, HER2, and Ki-67 expression in primary and recurrent/metastatic lesions in patients with primary early-stage breast cancer to obtain more evidence for clinical practice.

## Patients and Methods

The present study was a retrospective case series analysis. The study only involved the collection or review of existing data or diagnostic specimens collected anonymously. The protocol of this study was approved by The Institutional Review Board of Peking Union Medical College Hospital (PUMCH) (No. S-K1306). The need for informed consent was waived owing to the retrospective nature of the study.

### Patients

We retrospectively reviewed the medical records of patients with breast cancer treated at PUMCH (an academic teaching hospital with 2000 beds and over 3 million outpatients each year) from January 2005 to August 2018. The inclusion criteria were as follows: 1) age ≥18 years, 2) pathologically diagnosed with stage I or II breast cancer, and 3) presented with recurrent or metastatic breast cancer during the follow-up period after systemic therapy. The exclusion criteria were as follows: 1) male gender, 2) bilateral breast cancer, and 3) no record of the pathological diagnosis for either the primary or recurrent/metastatic lesions.

A total of 75 patients were included in the study. Forty patients (53.4%) received mastectomy + axillary lymph node dissection and eight patients (10.6%) received mastectomy + sentinel lymph node biopsy. Thirteen patients (17.3%) received lumpectomy + axillary lymph node dissection and 14 patients (18.6%) received lumpectomy + sentinel lymph node biopsy. Three patients received reoperations for the primary cancer due to positive margins. All patients were operable at diagnosis, and only one patient received neoadjuvant chemotherapy. The puncture result for this patient was consistent with the surgical results but a pathologic complete response (pCR) was not achieved. Most patients received adjuvant chemotherapy. The interval between surgery and systemic therapy was 2–4 weeks for most patients.

In the present study, 56 patients (56/75, 74.7%) opted for chemotherapy. The regimens consisted of doxorubicin/epirubicin + cyclophosphamide, docetaxel + cyclophosphamide, paclitaxel/docetaxel + doxorubicin, and docetaxel + doxorubicin/epirubicin + cyclophosphamide for HER2-negative patients. Docetaxel + cyclophosphamide + trastuzumab and doxorubicin + cyclophosphamide followed by docetaxel + trastuzumab were used for HER2-positive patients. The drug doses were doxorubicin/epirubicin 50/75 mg/m^2^, cyclophosphamide 500 mg/m2, paclitaxel/docetaxel 175/75 mg/m^2^, and trastuzumab 8 mg/kg IV on day 1 followed by 6 mg/kg every 3 weeks.

Twenty-six patients (26/75, 34.7%) received radiotherapy. For postmastectomy therapy, patients were usually treated in the supine position with a customized immobilization device. The target volume included the chest wall and supraclavicular fossa, and the patients were treated with 45–50 Gy at 2 Gy/fx with 6 Mv-X rays from a linear accelerator. The planning target covered the entire mastectomy scar, flaps, surgical clips, and drain sites included in the treatment field, avoiding junctional overdose with the supraclavicular field. A 5 mm bolus was used for half the duration of radiotherapy. An electron boost could be used to bring the total scar dose to 60–66 Gy in high-risk patients. For breast conserving therapy, patients were usually treated in the supine position with a customized immobilization device. Whole-breast irradiation was delivered first with tangential opposed fields using 2-Gy daily fractions up to 50 Gy with 6 Mv-X rays from a linear accelerator. The planning target volume (PTV) covered the superior border at the manubriosternal joint and the inferior border at 1 cm below the inframammary line. The medial border was usually the midline of the sternum, and the lateral border was the midaxillary line, excluding the outermost 5 mm from the superficial skin surface. For regional nodal irradiation, the lower part of the ipsilateral axillary lymph node (LN) area was included in all cases. The upper part of the ipsilateral axillary LN area was also included when metastases to the LNs were present.

Fifty-one patients (51/75, 68.0%) with hormone receptor-positive breast cancer received endocrine therapy with tamoxifen, aromatase inhibitors, and goserelin for ovarian function suppression. The duration of adjuvant endocrine therapy planned for 34 patients was 5 years and that for seven patients with a high risk of recurrence was 5–7 years. The seven patients with a high risk of recurrence had recurrence/metastasis within 5 years after surgery, and their endocrine therapy was terminated afterward. These seven patients had two or three lymph nodes involved and the Ki-67 index was positive. Histopathologic parameters were available for two patients. The ER status for one patient changed from positive to negative, the PR status for two patients changed from positive to negative, the HER2 status for one patient changed from negative to positive, and the Ki-67 status changed from positive to negative. All primary tumors were Luminal B tumors, six were still Luminal B after recurrence/metastasis, and the subtype for one tumor was uncertain after recurrence/metastasis. Tamoxifen was administered at doses of 10 mg twice a day or 20 mg once a day. Aromatase inhibitors included letrozole, anastrozole, and exemestane. Letrozole was used at a dose of 2.5 mg once a day, anastrozole at 1 mg once a day, and exemestane at 25 mg once a day. Goserelin acetate sustained-release depot was injected at a dose of 3.6 mg once a month.

### Diagnostic Approaches and Data Collection

Histopathology was used as the gold standard for the diagnosis. Patients were classified as having anatomic stage I or II breast cancer in accordance with the 8^th^ American Joint Committee on *Cancer* (AJCC) cancer staging manual [16, 19]. Recurrent/metastatic lesions were defined as any local or regional recurrence and/or any distant metastases. All patients had histopathological records for both the primary and recurrent/metastatic lesions.

ER and PR staining were evaluated according to the American Society of Clinical Oncology/College of American Pathologists Guidelines [20]. Positive nuclear staining with any intensity observed in at least 1% of tumor cells was defined as ER/PR positivity. HER2 expression was scored as 0, 1+, 2+, and 3 + according to the American Society of Clinical Oncology/College of American Pathologists Guidelines [21]. HER2 status was considered positive if the immunohistochemical staining score was 3+ and negative if the score was 1 + or 0. If the immunohistochemical score was 2+, the HER2 gene amplification status was determined using chromogenic *in-situ* hybridization [22]. The Ki-67 index was quantified based on the spot with the highest intensity in the high-power field (400x) and 1,200–1,500 cells were counted. The Ki-67 antibody (Clone SP6; Thermo Scientific, Japan) was used to define the Ki-67 status. A positive status for Ki-67 was defined as more than 14% of cells with Ki-67 antigen-antibody reactions counted under high-power magnification (×400) [23, 24]. The discordance rate for each biomolecular parameter was calculated as the frequency of changes between the primary tumors and the recurrent/metastatic lesions. The tumor subtypes were classified as Luminal A (ER and PR positive, HER2 negative, “low” Ki-67, and a “low” recurrence risk based on multi-gene-expression assay results if available), Luminal B (“Luminal B-like (HER2 negative)”: ER positive, HER2 negative, and at least one of the following: “high” Ki-67, “negative or low” PR, or “high” recurrence risk based on multi-gene-expression assay if available. “Luminal B-like (HER2 positive)”: ER positive, HER2 over-expressed or amplified with any Ki-67, and any PR) [25], HER2+ (Hormone receptor-negative and HER2-positive), and triple negative (TN) (Negative ER, PR, and HER2) according to St. Gallen’s Guide in 2013 [25]. There were differences in the reagent use and standardization process in different periods. These differences were minimized by repeated reviews, re-staining, and interpretation of questionable pathological results based on the principle of being faithful to the original results at the time.

The survival outcome was progression-free survival (PFS), which was defined as the time from the start of treatment (neoadjuvant therapies or surgery) to tumor progression or death [26]. The clinical characteristics of the patients were collected. The details of systemic treatment, recurrent or metastatic disease, and discordance in each biomolecular parameter between primary and recurrent/metastatic lesions as well as PFS were recorded. The patients were examined every six months within three years after the operation and once a year regularly after three years. Imaging examinations included: breast and axillary, abdominal, lymph nodes, and uterine and appendages ultrasound, chest CT, and bone scintigraphy (bone scintigraphy once a year for the first five years). Once the patient exhibited any symptoms, imaging examination was performed as soon as possible if necessary.

### Statistical Analyses

The continuous variables were presented as median with range, and categorical variables were presented as count and proportions. Fisher's exact test (or Pearson’s *chi*-square test) and the *t-*test were used to compare patient and tumor characteristics. Pearson’s *chi*-square test or Fisher’s exact and regression analyses were used to determine the relationship between discordance and any categorical variables. Survival curves were plotted with the Kaplan–Meier method, and differences in the survival curves were analyzed with the log-rank test. We set *p* < 0.05 as the threshold for statistical significance. All statistical analyses were performed using the software package SSPS 20.0 (SPSS Inc., United States).

## Results

### Patient Characteristics and Follow-Up Results

We reviewed the consecutive pathology findings for 8,691 women with breast cancers diagnosed between January 2005 and August 2018 at our hospital. A total of 133 patients were diagnosed with recurrent or metastatic tumors based on histopathology results. Of these, 75 patients had stage I or II disease, 31 had stage III or IV disease, and 27 patients could not be staged. For these 75 patients with stage I or II disease, the range of BMI was from 18.4 to 35.5. The number of patients who had a family history of breast cancer, ovarian cancer, pancreas cancer, and other cancers was 4, 1, 0, and 5, respectively. The most common comorbidity was hypertension. Other complications included hyperthyroidism, diabetes and coronary heart disease. Fifteen patients were postmenopausal when diagnosed with breast cancer, but none of them were treated with hormone replacement therapy.

The clinical characteristics of the included patients are shown in Table 1. The median age at diagnosis for all 75 patients was 44, ranging from 23 to 88 years old. In addition to ductal carcinoma and lobular carcinoma, there were 12 patients with special types of carcinoma, including mucinous carcinoma, micropapillary carcinoma, cribriform carcinoma, and two types of mixed carcinoma. The median pathological size for the maximum diameter of the included primary lesions was 2.0 cm, ranging from 0.1 to 4.5 cm. The median follow-up was 57.2 months (range 7.2 to 163.0 months). The median time after surgery before recurrence/metastasis was 37.0 months (range 3.1–163.5 months). Based on the immunohistochemical results for recurrent or metastatic tumors, patients were treated accordingly (see details in Table 1).

**TABLE 1:** Patient and tumor characteristics at primary diagnosis and recurrence/metastasis.

Patient characteristics (N = 75)	At primary diagnosis ^a^n (%)	At recurrence/metastasis ^a^n (%)
Age (years), median (range)	44 (23–88)	48.9 (25–92)
Tumor size (cm), median (range)	2.0 (0.1–4.5)	n/a
Body mass index, range	18.4–35.5	n/a
Stage at diagnosis		n/a
I	48 (64.0)	
II	27 (36.0)	
Menopausal status		
Premenopausal	58 (77.3)	55 (73.3)
Postmenopausal	15 (20.0)	18 (24.0)
Unknown	2 (2.7)	2 (2.7)
Pathological types		
Ductal	59 (78.7)	64 (85.3)
Lobular	4 (5.3)	4 (5.3)
Other	12 (16.0)	7 (9.3)
Histology grade		
1	4 (5.3)	7 (9.3)
2	43 (57.3)	48 (64.0)
3	28 (37.3)	17 (22.7)
Unknown	0 (0.0)	3 (4.0)
Number of pathologically positive lymph nodes		n/a
0	53 (70.7)	
1	21 (28.0)	
2	1 (1.3)	
Histopathologic parameters		n/a
Tumor-free margins	5 (6.7)	
Perinodal invasion	1 (1.3)	
Necrosis	1 (1.3)	
Vascular invasion	8 (10.7)	
Perineural invasion	1 (1.3)	
Cutaneous involvement	2 (2.7)	
Therapy		
Surgery	75 (100.0)	73 (97.3)
Chemotherapy	56 (74.7)	46 (61.3)
Radiotherapy	26 (34.7)	32 (42.7)
Endocrine therapy	51 (68.0)	26 (34.7)
Anti-HER2 therapy	8 (10.7)	8 (10.7)
Sites of biopsy	n/a	
Locoregional recurrence		68 (90.7)
Local		55
Lymph node		13
Distant metastasis		7 (9.3)
Liver		2
Lung		1
Bone		3
Distant skin		1
Histological sample for recurrence/metastasis	n/a	
Core puncture		4 (5.3)
Surgical resection		71 (94.7)
Estrogen receptor		
Positive	47 (62.7)	46 (61.3)
Negative	28 (37.3)	29 (38.7)
Progesterone receptor		
Positive	46 (61.3)	39 (52.0)
Negative	29 (38.7)	36 (48.0)
HER2		
Positive	15 (20.0)	20 (26.7)
Negative	57 (76.0)	51 (68.0)
Uncertain	3 (4.0)	4 (5.3)
Ki-67 index		
Positive	55 (73.3)	58 (77.3)
Negative	16 (21.3)	11 (14.6)
Unknown	4 (5.3)	6 (8.0)
Time after surgery before recurrence/metastasis (months), median (range)	n/a	37.0 (3.1–163.5)
<2 years		33 (44.0)
2–5 years		27 (36.0)
≥5 years		15 (20.0)
Death during the follow-up	2 (2.6)	

^a^ Or specified.

Abbreviation: ER, estrogen receptor; PR, progesterone receptor; HER2, human epidermal growth factor 2.

The positive expression results for ER, PR, HER2, and Ki-67 expression in the primary and recurrent or metastatic tumors can be found in Table 1. There was no significant statistical difference in these parameters between the primary tumors and the recurrent or metastatic tumors (*p* > 0.05).

The shortest interval between surgery and recurrence was 3.1 months, during which the patient underwent modified radical mastectomy after neoadjuvant chemotherapy and developed chest wall recurrence. Two patients died during the follow-up period; one died of brain metastasis and the other died of multiple-organ metastasis.

### Differences in Single Biomarker

Changes in the ER status were observed in seven patients (9.3%), with decreased expression (from a positive to negative status) in four patients and increased expression (from a negative to positive status) in three patients (Table 2). Differences in the PR status were found in 11 patients (14.7%), with decreased PR expression being the most common change (9/11). HER2 alterations were found in 10 patients (10/68, 14.7%), with decreased expression in three patients and increased expression in seven patients. Changes in Ki-67 were observed in 14 patients (14/65, 21.5%), with decreased expression in five patients and increased expression in nine patients. The status of ER, PR, HER2, and Ki-67 expression in the primary tumors and the recurrent or metastatic tumors was identical in only 33 patients.

**TABLE 2:** Differences in ER, PR, HER2, and Ki-67 expression between primary tumor and recurrent/metastatic lesions.

Biomarkers (n)^a^	Concordance n (%)	Discordance n (%)		
		Negative-positive	Positive-negative	Total
ER (75)	68 (90.7)	3	4	7 (9.3)
PR (75)	64 (85.3)	2	9	11 (14.7)
HER2 (68)	58 (85.3)	7	3	10 (14.7)
Ki-67 index (65)	51 (78.5)	9	5	14 (21.5)

^a^ The number of patients available for the records of the biomarkers.

Abbreviations: ER, estrogen receptor; PR, progesterone receptor; HER2, human epidermal growth factor 2.

If 10% was considered the threshold for ER and PR positivity, six results would change from the original positive determination to negative. The discordance rates of ER and PR expression were 10.6% and 18.6%, respectively.

### Differences in Tumor Subtypes

Overall, the subtype of the recurrent/metastatic lesions was different in 11 patients (11/64, 17.2%) (Table 3). The highest discordance rate (66.7%) was observed in patients with Luminal A primary tumors. The recurrent/metastatic lesions changed to the Luminal B subtype in three of these patients and to the TN subtype in one patient. On the contrary, TN primary tumors were the most stable, as no change from the TN subtype to other subtypes was observed. Patients with Luminal B and HER2 primary tumors had discordance rates of 11.8% and 14.3%, respectively.

**TABLE 3:** Changes in subtype between primary tumor and recurrent/metastatic lesions.

		Recurrent/metastatic tumor, n					Discordance, n (%)
		Luminal A	Luminal B	HER2	TN	Total	
Primary tumor, n	Luminal A	3	5	0	1	9	6 (66.7)
	Luminal B	2	30	1	1	34	4 (11.8)
	HER2	0	1	6	0	7	1 (14.3)
	TN	0	0	0	14	14	0 (0)
	Total	5	36	7	16	64	11 (17.2)

Abbreviations: HER2, human epidermal growth factor 2; TN, triple negative.

### Predictive Factors of Receptor Changes

The relationships between the differences in each biomolecular parameter and the potential predictive factors are shown in Table 4. There were statistical differences between the Luminal A subgroups in primary tumors and discordance (*p* < 0.05). No significant differences (*p* > 0.05) in any other biomarkers or tumor subtypes were observed for the potential predictors evaluated (e.g., primary tumor characteristics and therapies).

**TABLE 4:** Discordant proportion of ER, PR, HER2, and Ki-67 index according to potential predictors.

Primary tumor characteristics and therapies		Any discordance						
		ER, n (%)	PR, n (%)	HER2, n (%)	Ki-67, n (%)	Luminal A subtype, n (%)	Luminal B, n (%)	HER2 subtype, n (%)
ER+	Yes (n = 47)	4 (8.5)	11 (23.4)	9 (19.1)	11 (23.4)	6 (12.8)	3 (6.4)	1 (2.1)
	No (n = 28)	3 (10.7)	0	1 (3.6)	3 (10.7)	0	1 (3.6)	0
	*p* value	1^a^	0.015	0.117	0.228	0.078^a^	1^a^	1^a^
PR+	Yes (n = 46)	5 (10.9)	9 (19.6)	9 (19.6)	11 (23.9)	6 (13.0)	2 (4.3)	0
	No (n = 29)	2 (6.9)	2 (6.9)	1 (3.4)	3 (10.3)	0	2 (6.9)	1 (3.4)
	*p* value	0.7^a^	0.24	0.099	0.224	0.076^a^	0.638^a^	0.387^a^
HER2+	Yes (n = 15)	1 (6.7)	3 (20.0)	3 (20.0)	4 (26.7)	0	1 (6.7)	1 (6.7)
	No (n = 57)	6 (10.5)	8 (14.0)	7 (12.3)	9 (15.8)	6 (10.5)	3 (5.3)	0
	*p* value	1	0.867	0.727	0.55	0.333^a^	1^a^	0.208^a^
Ki-67+	Yes (n = 55)	3 (5.5)	8 (14.5)	7 (12.7)	9 (16.4)	4 (7.3)	4 (7.3)	1 (1.8)
	No (n = 16)	2 (12.5)	2 (12.5)	2 (12.5)	5 (31.3)	1 (6.3)	0	0
	*p* value	0.314^a^	1	1	0.337	1^a^	0.568^a^	1^a^
Luminal A subtype	Yes (n = 10)	2 (20.0)	2 (20.0)	1 (10.0)	3 (30.0)	6 (60.0)	0	0
	No (n = 60)	4 (6.7)	8 (13.3)	9 (15.0)	10 (16.7)	0	4 (6.7)	1 (1.7)
	*p* value	0.202^a^	0.944	1	0.572	0.000002	1^a^	1^a^
Luminal B subtype	Yes (n = 39)	3 (7.7)	8 (20.5)	9 (23.1)	7 (17.9)	0	4 (10.3)	0
	No (n = 31)	3 (9.7)	2 (6.5)	1 (3.2)	6 (19.4)	6 (19.4)	0	1 (3.2)
	*p* value	1^a^	0.185	0.044	0.881	0.006^a^	0.124^a^	0.443^a^
HER2 subtype	Yes (n = 7)	1 (14.3)	0	0	2 (28.6)	0	0	1 (14.3)
	No (n = 63)	5 (7.9)	10 (15.9)	10 (15.9)	11 (17.5)	6 (9.5)	4 (6.3)	0
	*p* value	0.482^a^	0.582^a^	0.582^a^	0.838	1^a^	1^a^	0.1^a^
TN subtype	Yes (n = 14)	0	0	0	1 (7.1)	0	0	0
	No (n = 56)	6 (10.7)	10 (17.9)	10 (17.9)	12 (21.4)	6 (10.7)	4 (7.1)	1 (1.8)
	*p* value	0.455	0.2	0.2	0.398	0.337	0.577^a^	1^a^
Histopathologic parameters	Yes (n = 18)	3 (16.7)	3 (16.7)	3 (16.7)	3 (16.7)	0	0	1 (5.6)
	No (n = 57)	4 (7.0)	8 (14.0)	7 (12.3)	11 (19.3)	6 (10.5)	4 (7.0)	0
	*p* value	0.446	1	0.937	1	0.326^a^	0.567^a^	0.24^a^
Relapse site	LR (n = 68)	7 (10.3)	9 (13.2)	8 (11.8)	13 (19.1)	6 (8.9)	3 (4.4)	1 (1.4)
	DM (n = 7)	0	2 (28.6)	2 (28.6)	1 (14.3)	0	1 (14.3)	0
	*p* value	1^a^	0.595	0.233^a^	1^a^	1^a^	0.330^a^	1^a^
Chemotherapy	Yes (n = 56)	5 (8.9)	10 (17.9)	8 (14.3)	11 (19.6)	4 (7.1)	3 (5.4)	1 (1.8)
	No (n = 19)	2 (10.5)	1 (5.3)	2 (10.5)	3 (15.8)	2 (10.5)	1 (5.3)	0
	*p* value	1	0.334	0.979	0.975	0.64^a^	1^a^	1^a^
Radiotherapy	Yes (n = 26)	4 (15.4)	2 (7.7)	2 (7.7)	3 (11.5)	0	1 (3.8)	0
	No (n = 49)	3 (6.1)	9 (18.4)	8 (16.3)	11 (22.4)	6 (12.2)	3 (6.1)	1 (2.0)
	*p* value	0.371	0.368	0.490	0.399	0.087^a^	1^a^	1^a^
Endocrine therapy	Yes (n = 51)	6 (11.8)	11 (21.6)	9 (17.6)	12 (23.5)	6 (11.8)	3 (5.9)	0
	No (n = 24)	1 (4.2)	0	1 (4.2)	2 (8.3)	0	1 (4.2)	1 (4.2)
	*p* value	0.803	0.126	0.42	0.476	0.328^a^	1^a^	0.253^a^
Anti-HER2 therapy	Yes (n = 8)	1 (12.5)	0	1 (12.5)	2 (25.0)	0	1 (12.5)	1 (12.5)
	No (n = 67)	6 (9.0)	11 (16.4)	9 (13.4)	12 (17.9)	6 (9.0)	3 (4.5)	0
	*p* value	0.562^a^	0.476	1	0.995	1^a^	0.369^a^	0.107^a^

^a^ Fisher's test; Abbreviations: ER, estrogen receptor; PR, progesterone receptor; HER2, human epidermal growth factor 2; LR, locoregional recurrence, DM, distant metastasis.

### Clinical Impact

At the primary diagnosis, three patients in the HER2-positive group did not receive anti-HER2 therapy because the tumor was less than 1 cm with negative lymph nodes and four patients did not receive the therapy because of economic or personal reasons. After recurrence, eight patients did not receive anti-HER2 therapy because of chest wall recurrence and four did not receive the therapy because of economic or personal reasons.

Among 18 patients (24.0%, 18/75) with ER/PR expression changes and 10 patients (13.3%, 10/75) with HER2 expression changes, treatments based on the recurrent receptor status were applied for 25 patients (89.3%, 25/28). The treatment for three out of five patients with ER or PR changes from negative to positive was adjusted to endocrine therapy; three received additional chemotherapy and one received additional radiotherapy. Four out of 13 patients with ER or PR changes from positive to negative received chemotherapy after biopsy, eight discontinued endocrine therapy, and one received additional radiotherapy. One patient with HER2 change from negative to positive received anti-HER2 treatment, three received additional endocrine treatment, and two received additional radiotherapy. The treatment for 69 patients was altered after recurrence or metastasis and 66 of these patients remained recurrence-free after resection of the recurrent lesions.

### Survival Analysis

**FIGURE 1:**
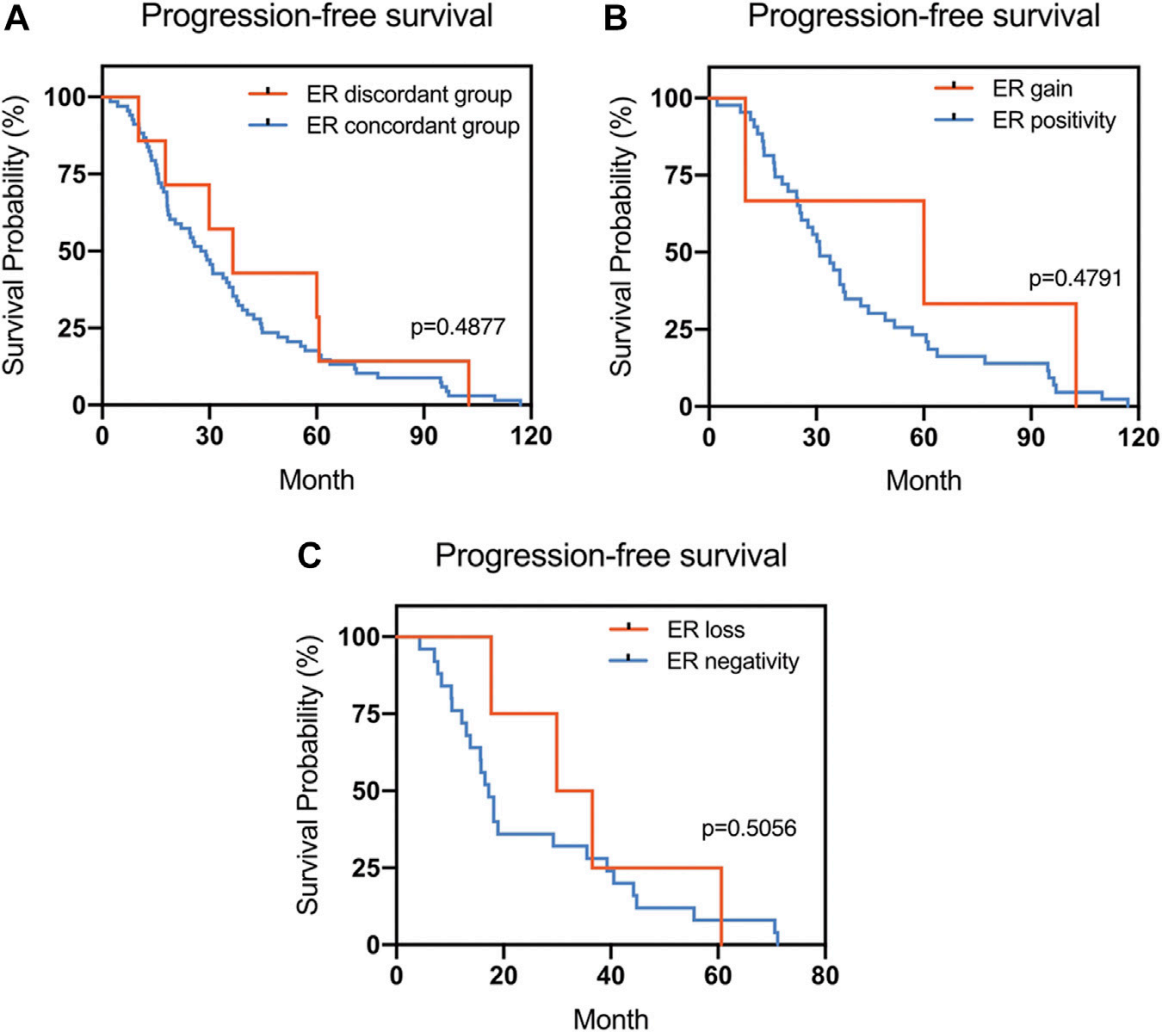
Kaplan–Meier survival curves for progression-free survival (PFS) in patients with recurrent/metastatic breast cancer. **(A)** Stratified for ER concordance vs. discordance. **(B)** Stratified for ER change from negative to positive vs. unchanged ER-positivity. **(C)** Stratified for ER change from positive to negative vs. unchanged ER-negativity.

**FIGURE 2:**
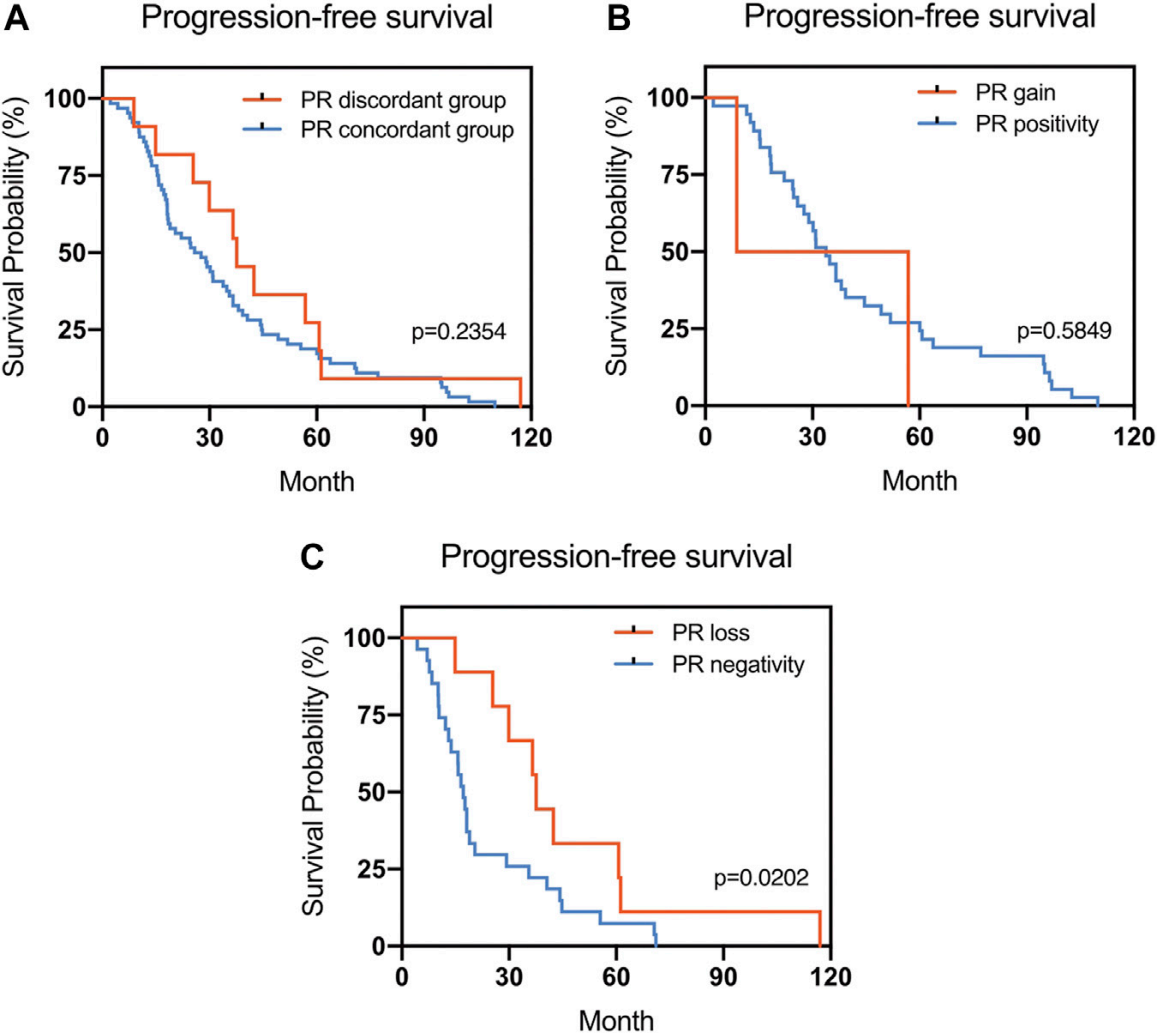
Kaplan–Meier survival curves for progression-free survival (PFS) in patients with recurrent/metastatic breast cancer. **(A)** Stratified for PR concordance vs. discordance. **(B)** Stratified for PR change from negative to positive vs. unchanged PR-positivity. **(C)** Stratified for PR change from positive to negative vs. unchanged PR-negativity.

**FIGURE 3:**
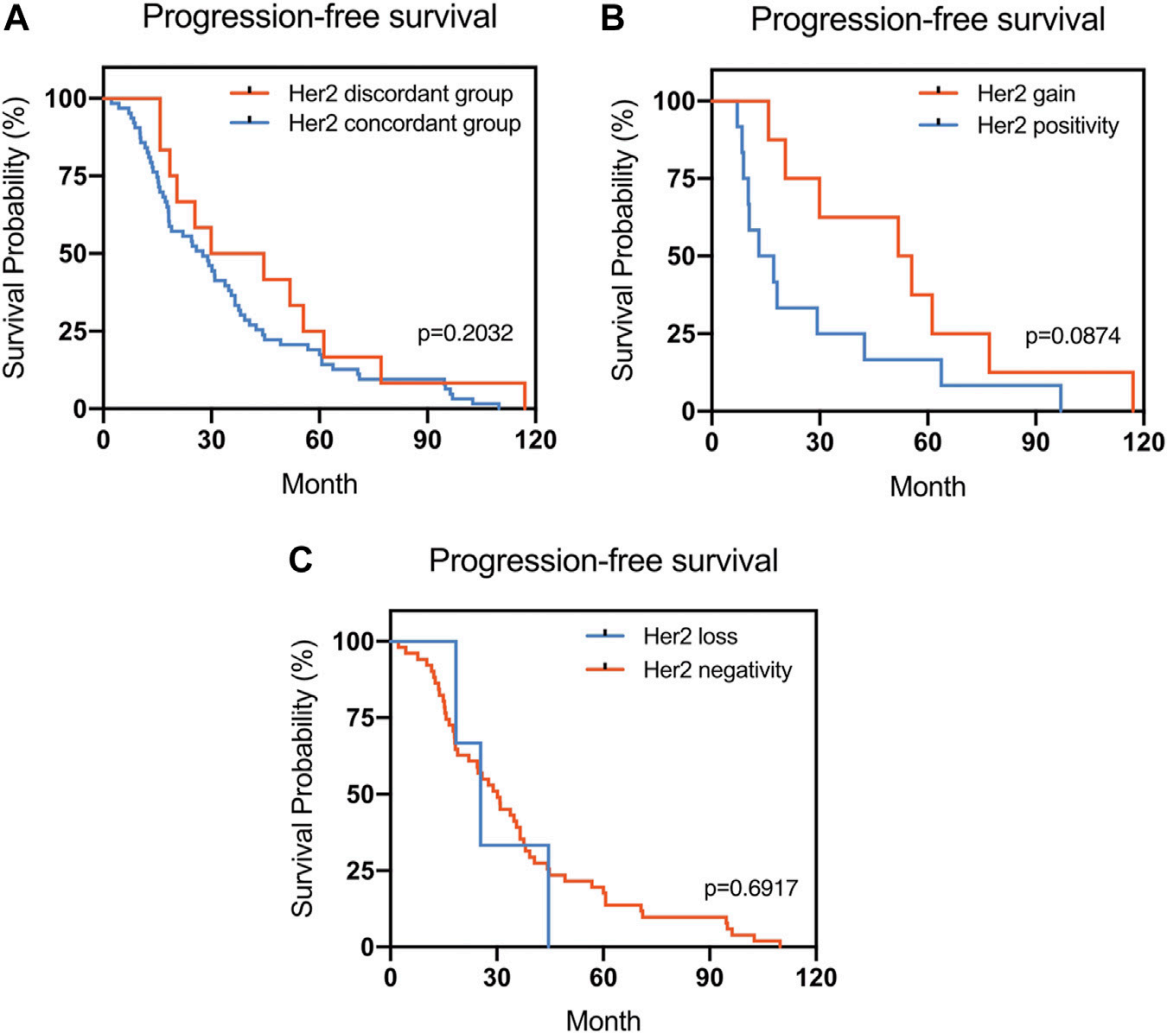
Kaplan–Meier survival curves for progression-free survival (PFS) in patients with recurrent/metastatic breast cancer. **(A)** Stratified for HER2 concordance vs. discordance. **(B)** Stratified for HER2 change from negative to positive vs. unchanged HER2-positivity. **(C)** Stratified for HER2 change from positive to negative vs. unchanged HER2-negativity.

**FIGURE 4:**
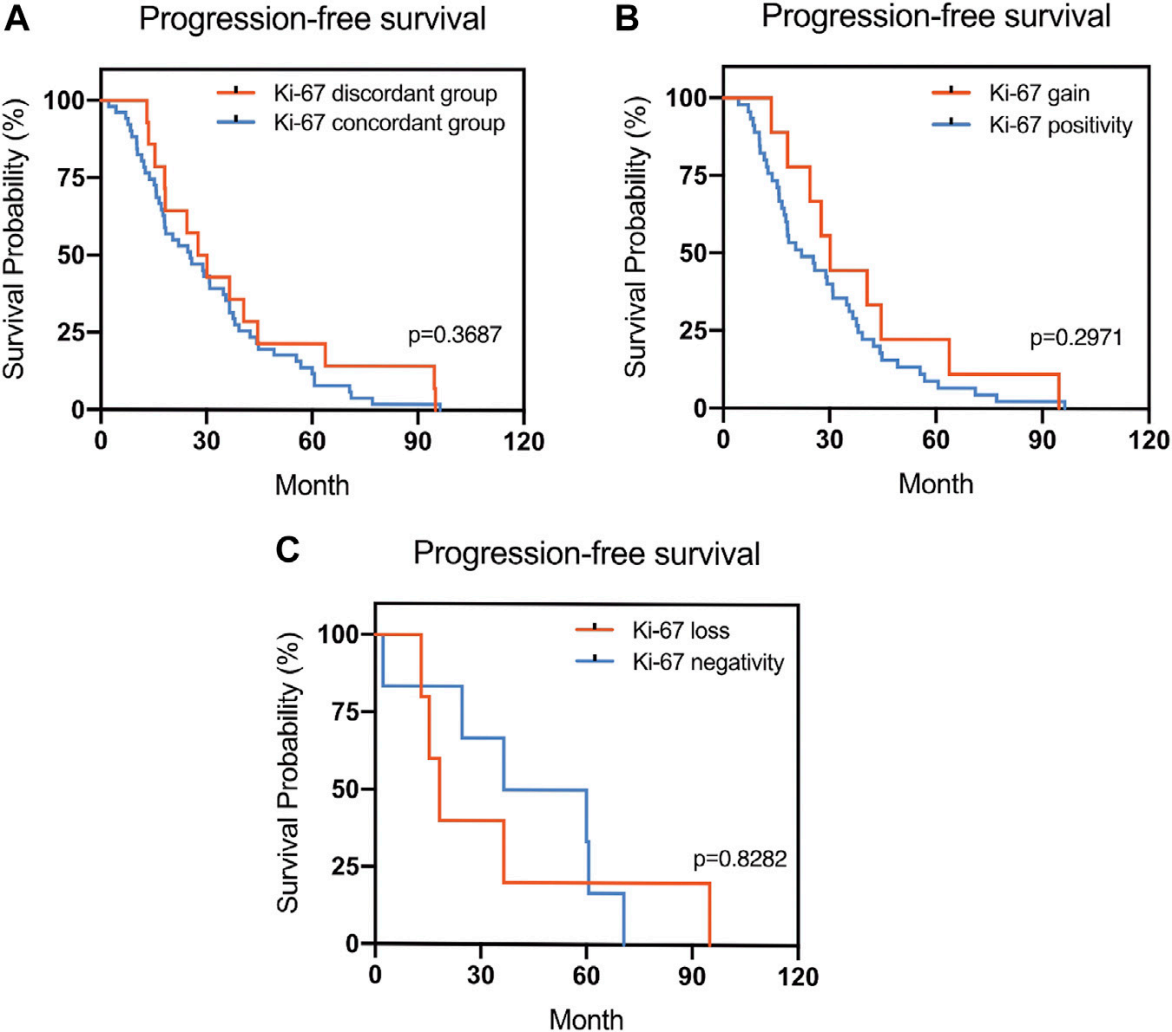
Kaplan–Meier survival curves in patients with recurrent/metastatic breast cancer for progression-free survival (PFS). **(A)** Stratified for Ki-67 concordance vs. discordance. **(B)** Stratified for Ki-67 change from negative to positive vs. unchanged Ki-67-positivity. **(C)** Stratified for Ki-67 change from positive to negative vs. unchanged Ki-67-negativity.

The Kaplan–Meier survival curves for PFS in the patients can be found in Figures 1–4. The PFS was compared according to the discordance vs. accordance of the receptors, negative to positive (gain) vs. an unchanged positive result for the receptor, and positive to negative (loss) vs. an unchanged negative result for the receptor. A significant difference was found between patients with PR loss and those with an unchanged negative PR result (*p* = 0.020). There was no statistically significant difference in any of the other parameters evaluated (*p* > 0.05).

**FIGURE 5:**
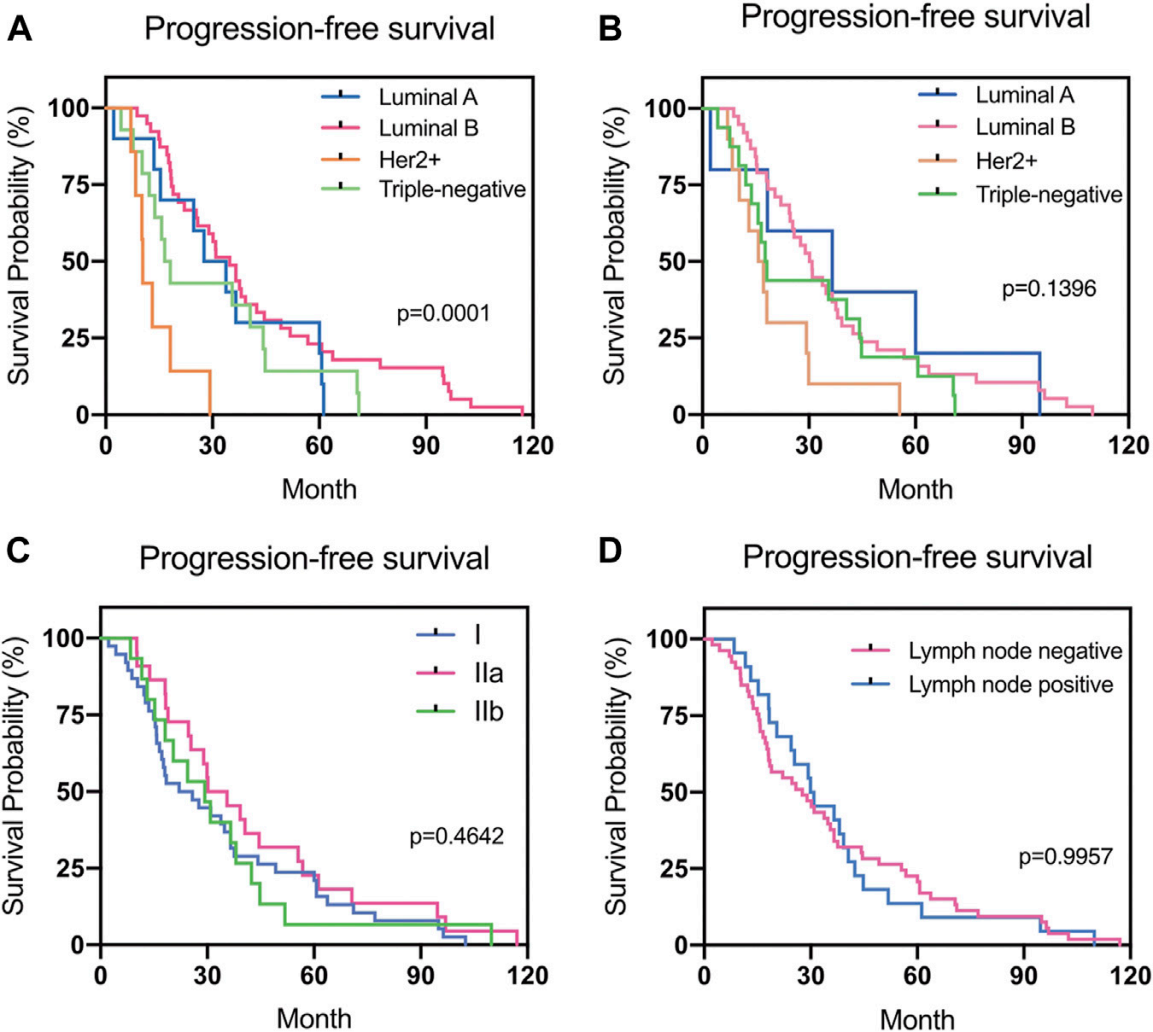
Progression-free survival (PFS) in different groups. **(A)** Relationship between PFS and the subtype of primary breast cancer. **(B)** Relationship between PFS and the subtype of recurrent or metastatic breast cancer. **(C)** Relationship between PFS and the stage of primary breast cancer. **(D)** Relationship between PFS and the lymph node status during surgery.

There was a statistical difference in the PFS of patients according to the subtype of the primary breast cancer (*p* = 0.0001). However, no statistical difference in PFS according to the subtype of the recurrent or metastatic breast cancer was found (*p* > 0.05) (Figure 5).

## Discussion

In the present study, we evaluated 75 patients with primary early stage breast cancer who developed recurrence or metastasis after systemic therapy and no relapses developed under chemotherapy or radiotherapy. Differences in ER, PR, HER2, and Ki-67 expression between the primary and recurrent/metastatic tumors were observed in 9.3%, 14.7%, 14.7%, and 21.5% of patients, respectively. Luminal A, Luminal B, HER2, and TN primary tumors changed to a different subtype in 66.7%, 11.8%, 14.3%, and 0% of patients, respectively. According to the regression analysis results, no significant factors predicted the changes observed. Among 69 patients whose treatments were modified after recurrence or metastasis, 66 patients remained recurrence-free after resection of the recurrent lesions. Changes in most of the evaluated receptors did not impact the PFS of the patients, except among patients with PR loss, who had better PFS (*p* = 0.020) than those with unchanged negative PR results. Although there was a statistical difference in PFS according to the subtype of the primary breast cancer (*p* = 0.0001), no statistical difference in PFS according to the subtypes of recurrent or metastatic breast cancer was found (*p* > 0.05).

The discordance of ER, PR, and HER2 expression between primary tumors and recurrent or metastatic tumors have been widely reported and theories regarding the mechanism underlying these changes include the variability of detection performance, tumor heterogeneity, the organ microenvironment, and biological evolution [27–29]. Another hypothesis is that a small number of cancer cells are prone to recurrence or metastasis and these cells may not be detectable at the initial diagnosis of primary breast cancer [27, 30].

Several previous studies have reported differences in the biomolecular characteristics of primary breast cancer and recurrent/metastatic lesions ([12, 31–38]). In the present study, the discordance rate for ER expression was 9.3% (7/75), which was slightly lower than that (10.3–30.4%) reported for patients with all-stage breast cancer in previous studies. Earlier reports of the discordant rate for PR expression ranged from 16.0% to 54.3%, which is much higher than the 14.7% in our cohort (11/75). However, our results for the HER2 receptor (14.7%, 10/68) are similar to those reported in previous studies (8.3–27.0%). In our study, the hormone receptor status for more than 85% of patients with early stage breast cancer remained the same, suggesting the initial endocrine therapy will continue to work.

Different molecular subtypes for breast cancer have different prognoses [39, 40]. In the present study, 11 patients (11/64, 17.2%) presented with different subtypes for the recurrent/metastatic lesions. TN primary tumors were the most consistent (accordance rate 100%), whereas the discordant rate for Luminal A tumors was the highest (66.7%). The high rate of discordance in Luminal A tumors was primarily due to reduced ER or PR expression and changes in the HER2 and Ki-67 expression from a negative to a positive status when recurrence or metastasis occurred. The subtype changes observed in this study were similar to those in previous studies [12, 31]. Breast cancer is a heterogeneous disease and understanding the stratification within the tumor is paramount to achieving better clinical outcomes [41]. The genomic heterogeneity of metastatic breast cancer explains why the receptor status measured exclusively in the primary tumor may not be sufficiently informative for predicting the patient's responsiveness to therapy [42]. We are convinced that a better understanding of the composition and evolution of tumors in the course of progression and treatment can improve patient management [43]. In this study, PFS was lowest among patients with HER2-positive primary breast cancer. However, although there was a statistical difference in the PFS according to the subtype of the primary breast cancer (*p* = 0.0001), no statistical difference in the PFS according to the subtype of recurrent or metastatic breast cancer was found (*p* > 0.05). The results may be due to the adaptation of treatment over time according to changes in the molecular receptors.

Locoregional relapse is biologically different from distant relapse for breast cancer. In the present study, we only had seven cases of distant metastases. The highest ratio of “any discordance” in PR and HER2 expression was found in the distant metastases group. Due to the limited number of distant metastases, we did not perform subgroup analysis accordingly. However, the difference between locoregional relapse and distant metastases on the topic is worth exploring in the future.

Researchers are still investigating the possible reasons (e.g., genetic heterogeneity, chemoradiotherapy effect, etc.) for differences in receptor expression between primary and recurrent/metastatic tumors [38, 44–46]. Several researchers have proposed potential predictors (e.g., chemotherapy, relapse site, etc.) for these differences ([9, 13, 14, 37]). However, no confirmed conclusion has been reached to date. In our analysis, no specific factor was identified in the 75 patients that could predict changes in the expression of any of the receptors evaluated (*p* > 0.05). Studies with a larger scale are needed to fill the knowledge gap in the future.

There are different guidelines for the management of breast cancer recurrence or metastasis, but all guidelines recommend confirming the receptor status of the tumor [38, 47–49]. In practice, it is difficult to obtain the receptor information for recurrent and metastatic lesions primarily due to a lack of willingness and the physical condition of the patient, the location of metastasis, and technical limitations [50]. Among 69 patients for whom the treatment was altered after recurrence or metastasis, 66 patients remained recurrence-free after resection of the recurrent lesions. Therefore, we suggest obtaining information on recurrent or metastatic tumors by biopsy and puncture. This ensures an accurate determination of recurrent or metastatic disease and tumor histology and allows for biomarker determination as well as the selection of appropriate treatment [12, 27].

At present, ER, PR, and HER2 status is assessed in primary breast carcinomas as part of the standard clinical procedures. However, various treatments affect the residual tumor cells in different ways. Herein, whole-genome sequence data suggest that disseminated tumor cells arise from sub-clonal populations of cells in the primary tumor and undergo further sequence evolution after dissemination [42, 51, 52]. In the course of disease development, biomarkers can change, genetic variation may occur, and significant molecular differences with therapeutic implications may appear between primary tumors and the circulating tumor cells [53, 54]. What is believed to be an “intrinsic subtype” also seems dynamic or, more precisely, pseudo-dynamic, and classifications based on immunohistochemistry are also similarly influenced [55]. Identification of the most appropriate strategies and their implementation in the clinic will prove highly challenging and necessitate the adoption of radical new practices for the optimal clinical management of breast malignancies. DNA microarrays and next-generation sequencing are currently providing valuable insight into intratumor and intratumor heterogeneity [56]. Further clinical studies on the genomic level should be initiated for early-stage breast cancer patients with recurrence and metastasis.

To our knowledge, this is one of the largest studies conducted in one research center to evaluate early-stage breast cancer patients with recurrence or metastasis after systemic therapy and differences in immunohistochemical markers between the primary tumor and recurrent or metastatic tumors. However, there are several limitations. First, there is selection and information bias in this study due to the long-term retrospective nature. Second, because the study was performed in one hospital, the external validity is limited. Third, although we differentiated between new ipsilateral primary and recurrent tumors using the pathological morphology, grade, and subtypes, the accuracy could not be confirmed by genomic hybridization. This was a limitation and a possible explanation for mismatches. Lastly, although this is the most extensive study comparing differences between primary and recurrent/metastatic tumors in early stage breast cancer patients at one center in China, the sample size of 75 may not have enough power for all types of analyses. A future multicenter study with a large scale on this topic is necessary.

For patients with early-stage breast cancer, the ER, PR, HER2, and Ki-67 expression profile for recurrence/metastasis does not always match that of the primary tumor. After adjusting treatment according to the receptor status of recurrent/metastatic lesions, most patients remained progression-free during the follow-up period. Therefore, the reassessment of the biomolecular status of recurrent/metastatic tumors and appropriate modification of the treatment strategy is suggested.

## Data Availability

The raw data supporting the conclusions of this article will be made available by the authors, without undue reservation.
